# Admission Serum Total Brain-Derived Neurotrophic Factor and Angiographic No-Reflow in Non-ST-Segment Elevation Myocardial Infarction Undergoing Percutaneous Coronary Intervention

**DOI:** 10.3390/medicina62071211

**Published:** 2026-06-23

**Authors:** Alp Yıldırım, Mustafa Çelik, Müzeyyen Gizem Parmak, Muhammet Salih Ateş, Erdoğan Sökmen, Kenan Güçlü

**Affiliations:** 1 Department of Cardiology, Faculty of Medicine, Ahi Evran University, 40100 Kirsehir, Türkiye; alp.yildirim@ahievran.edu.tr (A.Y.); mgizemp@gmail.com (M.G.P.); erdoganmen@gmail.com (E.S.); 2 Department of Cardiology, Faculty of Medicine, Necmettin Erbakan University, 42090 Konya, Türkiye; muscelik50@gmail.com; 3 Department of Medical Biochemistry, Kayseri State Hospital, 38030 Kayseri, Türkiye; kguclu2001@gmail.com

**Keywords:** brain-derived neurotrophic factor (BDNF), non-ST-segment elevation myocardial infarction (NSTEMI), no-reflow (NRF), percutaneous coronary intervention, TIMI flow, myocardial blush grade, ROC analysis, internal validation

## Abstract

*Background and Objectives*: Angiographic no-reflow (NRF) after percutaneous coronary intervention (PCI) reflects impaired microvascular reperfusion despite successful treatment of the epicardial culprit lesion. Brain-derived neurotrophic factor (BDNF) is a neurotrophin involved in endothelial signaling, platelet biology, inflammation, and angiogenesis. Its relationship with NRF in non-ST-segment elevation myocardial infarction (NSTEMI) remains insufficiently characterized. *Materials and Methods*: This single-center prospective observational cohort study included 700 consecutive NSTEMI patients undergoing culprit-lesion PCI. Admission serum total BDNF was measured before PCI using a standardized enzyme-linked immunosorbent assay protocol. Angiographic NRF was defined as final thrombolysis in myocardial infarction (TIMI) flow <3 and/or TIMI 3 flow with myocardial blush grade (MBG) 0–1 in the absence of residual stenosis, dissection, severe spasm, or other mechanical obstruction. Four sequential logistic regression models were used to evaluate the stability of the association between BDNF and NRF: Model 1 adjusted for clinical variables; Model 2 further adjusted for laboratory and inflammatory variables; Model 3 further adjusted for cardiac injury and functional variables; and Model 4 further adjusted for angiographic and procedural variables. Discrimination, calibration, reclassification, decision-curve analysis, and internal validation were assessed. *Results*: NRF occurred in 114 of 700 patients (16.3%). Serum total BDNF was higher in the NRF group than in the reflow group [555 (465–688) vs. 386 (292–496) pg/mL, *p* < 0.001]. BDNF remained independently associated with NRF across sequential models: Model 1 OR 1.67 per 100 pg/mL (95% CI 1.43–1.96), Model 2 OR 1.49 (95% CI 1.24–1.79), Model 3 OR 1.41 (95% CI 1.16–1.72), and Model 4 OR 1.31 (95% CI 1.07–1.60). The BDNF-only AUC was 0.787, while the final BDNF-enriched Model 4 reached an AUC of 0.866. The optimism-corrected bootstrap AUC was 0.852 and the 10-fold cross-validated AUC was 0.845. *Conclusions*: Higher admission serum total BDNF was independently associated with angiographic NRF in NSTEMI patients undergoing PCI and improved risk discrimination when added to clinical, biochemical, cardiac, and angiographic predictors. These findings suggest that serum total BDNF may reflect a context-dependent biomarker signal related to acute thrombo-inflammatory, platelet-associated, and microvascular injury pathways; however, the observed incremental value was modest and requires external validation.

## 1. Introduction

Angiographic no-reflow (NRF) is defined as inadequate myocardial reperfusion after treatment of the epicardial culprit lesion. In clinical PCI practice, it is usually recognized by reduced final TIMI flow and/or impaired myocardial blush in the absence of a mechanical explanation such as residual stenosis, dissection, severe epicardial spasm, distal vessel cut-off, or equipment-related obstruction. Although NRF is most frequently discussed in STEMI, it also occurs in NSTEMI, particularly when thrombus burden, impaired baseline flow, lesion complexity, renal dysfunction, inflammation, and myocardial injury are present [1,2,3,4,5,6].

NRF is not a single-mechanism event. It results from the convergence of distal microembolization, endothelial swelling, capillary obstruction, neutrophil and platelet plugging, microvascular spasm, oxidative stress, and reperfusion injury [1,2,3,4]. This explains why classic clinical and angiographic variables do not fully capture individual susceptibility. A biomarker that integrates inflammation, platelet activation, endothelial stress, and microvascular vulnerability could provide useful incremental information before or during PCI.

Brain-derived neurotrophic factor (BDNF) is a member of the neurotrophin family. It is well known for its neuronal actions, but it also has cardiovascular relevance. BDNF is stored in platelets and released during platelet activation, is expressed in endothelial and vascular cells, may modulate TrkB-dependent endothelial survival, and may influence nitric oxide bioavailability, vascular tone, angiogenesis, and inflammatory signaling [7,8,9,10,11,12,13]. These features make BDNF biologically attractive in acute coronary syndromes, but they also make its interpretation context-dependent.

In chronic or non-occlusive microvascular conditions, low BDNF or low mature BDNF may indicate impaired endothelial repair and reduced vascular homeostasis. A recent study of coronary slow flow reported lower serum mature BDNF in patients with the coronary slow-flow phenomenon and an inverse association between mature BDNF and mean TIMI frame count [14]. In contrast, ACS-related studies suggest that high serum BDNF may reflect platelet release, culprit-plaque inflammation, thrombus activity, and acute tissue injury. Montone et al. reported higher serum BDNF in STEMI than in NSTE-ACS and showed an association between BDNF and OCT-defined macrophage infiltration in culprit plaques [15]. A recent STEMI/NRF study further suggested that BDNF levels were higher in patients with slow-flow/NRF after primary PCI and that BDNF may serve as an NRF-related biomarker in acute myocardial infarction [16].

The specific relationship between serum total BDNF and angiographic NRF in NSTEMI remains incompletely defined. Therefore, this study aimed to evaluate whether admission serum total BDNF is associated with angiographic NRF in NSTEMI patients undergoing culprit-lesion PCI and whether BDNF provides incremental predictive information beyond established clinical, biochemical, cardiac, angiographic, and procedural predictors.

## 2. Materials and Methods

### 2.1. Study Design and Patient Selection

This single-center prospective observational cohort study enrolled consecutive adult patients with ACS who underwent coronary angiography during the index hospitalization between December 2024 and May 2026. Among these patients, those diagnosed with non-ST-segment elevation myocardial infarction (NSTEMI) and treated with culprit-lesion percutaneous coronary intervention (PCI) were evaluated for inclusion. NSTEMI was defined according to contemporary guideline criteria as the presence of ischemic symptoms or an equivalent clinical presentation, elevated cardiac troponin with a rising and/or falling pattern, and absence of persistent ST-segment elevation [17,18].

The inclusion criteria were as follows: (1) age ≥ 18 years; (2) diagnosis of NSTEMI according to contemporary guideline criteria; (3) performance of coronary angiography during the index hospitalization; (4) identification of a culprit coronary lesion treated with PCI; and (5) availability of admission serum total brain-derived neurotrophic factor (BDNF) measurement before culprit-lesion PCI.

**Figure 1:**
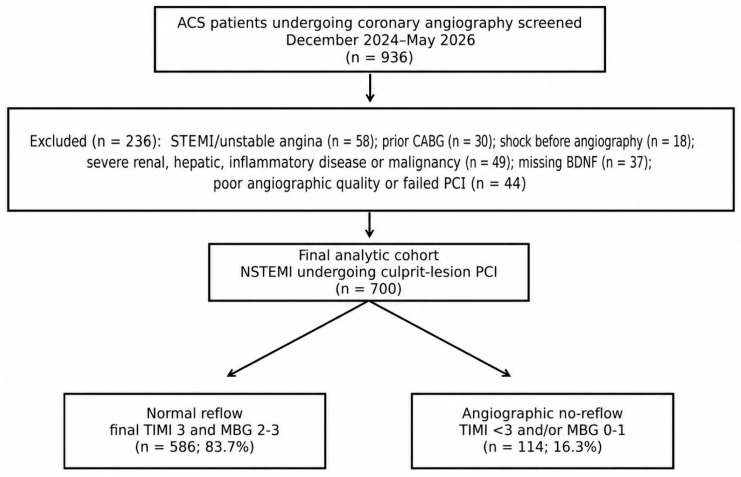
Study flowchart showing screening, exclusions, and grouping according to angiographic NRF status.

A total of 936 ACS patients undergoing coronary angiography were screened. Exclusion criteria included ST-segment elevation myocardial infarction, unstable angina without biomarker elevation, prior coronary artery bypass grafting, cardiogenic shock before angiography, active systemic infection, chronic inflammatory or autoimmune disease, known malignancy, severe hepatic dysfunction, dialysis-dependent renal failure, missing BDNF measurement, non-diagnostic coronary angiography, and failed culprit-lesion PCI. These exclusion criteria were selected to create a clinically homogeneous NSTEMI-PCI cohort, to avoid conditions that could independently alter circulating BDNF, inflammatory or platelet-related biomarkers, and to ensure reliable angiographic adjudication of post-PCI no-reflow. After application of these criteria, 700 patients were included in the final analysis (Figure 1).

The study protocol was approved by the Ahi Evran University Faculty of Medicine Clinical Research Ethics Committee (approval date: 10 December 2024; approval number: 2024-20/175) and was conducted in accordance with the principles of the Declaration of Helsinki. Written informed consent was obtained from all participants before inclusion in the study.

### 2.2. Serum Total BDNF Measurement

Peripheral venous blood samples for total brain-derived neurotrophic factor (BDNF) measurement were obtained on admission before coronary angiography and culprit-lesion PCI. Blood samples were collected into plain serum tubes and allowed to clot at room temperature for 30 min. The samples were then centrifuged at 3000× *g* for 15 min, and the separated serum was aliquoted and stored at −80 °C until biochemical analysis. Repeated freeze–thaw cycles were avoided.

Serum total BDNF concentrations were measured in duplicate using a commercially available enzyme-linked immunosorbent assay kit specific for human BDNF (Human BDNF ELISA Kit, R&D Systems, Minneapolis, MN, USA), in accordance with the manufacturer’s instructions. Absorbance values were read at 450 nm using a BioTek ELx800 microplate spectrophotometer (BioTek Instruments, Winooski, VT, USA). BDNF concentrations were calculated from standard calibration curves generated using the calibrators supplied with the kit. The intra-assay and inter-assay coefficients of variation were <8% and <10%, respectively. Laboratory personnel performing the assays were blinded to all clinical, procedural, and angiographic NRF data.

### 2.3. Clinical, Echocardiographic, and Angiographic Data

Demographic characteristics, cardiovascular risk factors, medical history, admission vital signs, laboratory variables, and medication use were extracted from institutional electronic records. Routine laboratory parameters were obtained from admission blood samples. High-sensitivity C-reactive protein (hs-CRP), white blood cell (WBC) count, neutrophil-to-lymphocyte ratio (NLR), platelet count, mean platelet volume (MPV), platelet distribution width (PDW), glucose, renal function, lipid variables, albumin, uric acid, and cardiac biomarkers were recorded when available.

Transthoracic echocardiography was performed within the first 24 h of admission. Left ventricular ejection fraction (LVEF) was calculated using the modified Simpson biplane method when image quality permitted. Coronary angiograms were reviewed by two experienced interventional cardiologists blinded to BDNF and routine laboratory data. Disagreements were resolved by consensus or by a third senior interventional cardiologist. Baseline TIMI flow, final TIMI flow, MBG, culprit vessel, thrombus burden, multivessel disease, lesion length, stent length, predilatation, post-dilatation, direct stenting, and bailout glycoprotein IIb/IIIa inhibitor use were recorded. The SYNTAX score was calculated using the original scoring principles. Interobserver agreement for angiographic NRF and high thrombus burden was assessed using Cohen’s kappa coefficient. Because MBG is an ordinal tissue-level reperfusion score and a co-defining component of the NRF endpoint, interobserver agreement for MBG grading was assessed using weighted kappa. Agreement for the SYNTAX score was evaluated using the intraclass correlation coefficient.

### 2.4. Definition of Angiographic No-Reflow

The primary endpoint was angiographic NRF after culprit-lesion PCI. NRF was defined as a final TIMI flow grade <3 and/or TIMI 3 flow with MBG 0–1, in the absence of flow-limiting residual stenosis, coronary dissection, severe epicardial spasm, embolized thrombus causing a new abrupt mechanical occlusion, or equipment-related obstruction. This definition captures both epicardial and tissue-level reperfusion impairment and is aligned with contemporary NRF concepts using TIMI flow and myocardial blush [1,2,3,4,19,20].

### 2.5. Statistical Analysis and Four-Model Regression Strategy

Continuous variables are expressed as mean ± standard deviation or median (interquartile range), depending on distribution. Categorical variables are presented as numbers and percentages. The independent-samples *t*-test or Mann–Whitney U test was used for continuous variables, and the chi-square or Fisher’s exact test was used for categorical variables. Spearman correlation was used to evaluate the relationship between serum BDNF and continuous or ordinal variables. Interobserver agreement for angiographic NRF diagnosis and high thrombus burden was evaluated using Cohen’s kappa coefficient, agreement for ordinal MBG grading was evaluated using weighted kappa, and agreement for SYNTAX score was assessed using the intraclass correlation coefficient. A two-sided *p* value < 0.05 was considered statistically significant.

BDNF was analyzed as a continuous variable per 100 pg/mL increase. To evaluate whether BDNF remained significant after sequential adjustment, four logistic regression models were prespecified. Model 1 included BDNF and clinical variables: age, male sex, diabetes mellitus, hypertension, smoking status, and body mass index. Model 2 included Model 1 plus laboratory and inflammatory variables: estimated glomerular filtration rate (eGFR), admission glucose, ln-transformed hs-CRP, WBC count, hemoglobin, and platelet count. Model 3 included Model 2 plus cardiac injury and functional variables: LVEF, ln-transformed peak high-sensitivity cardiac troponin I (hs-cTnI), Killip class ≥ II, and symptom-to-angiography time. Model 4 included Model 3 plus angiographic and procedural variables: baseline TIMI 0–1 flow, high thrombus burden, SYNTAX score, lesion length, and multivessel disease. BDNF was forced into all four models to evaluate the stability of the biomarker signal across adjustment layers.

The incremental predictive value of BDNF was evaluated by comparing Model 4 without BDNF versus Model 4 with BDNF. Discrimination was quantified using receiver-operating characteristic (ROC) curves and area under the curve (AUC) with 95% confidence intervals (CI). Pairwise comparisons of AUCs were performed using the DeLong test. The optimal BDNF threshold was estimated using the Youden index. Calibration was assessed using calibration intercept, calibration slope, Brier score, and the Hosmer-Lemeshow goodness-of-fit test. Reclassification was summarized by continuous net reclassification improvement (NRI) and integrated discrimination improvement (IDI). Decision-curve analysis was used to estimate clinical net benefit. Internal validation was performed with 1000 bootstrap resamples and 10-fold cross-validation, consistent with modern prediction-model reporting principles [21,22]. Covariates were prespecified according to clinical relevance, prior no-reflow literature, and biological plausibility to reduce post hoc model selection and overfitting. Missing covariate data were minimal; the primary models used multiple imputation by chained equations with 20 imputed datasets, and pooled estimates were obtained according to Rubin’s rules. Complete-case analysis was performed as a sensitivity analysis. Statistical analyses were performed using IBM SPSS Statistics version 26.0 (IBM Corp., Armonk, NY, USA) and R software version 4.3.2 (R Foundation for Statistical Computing, Vienna, Austria).

## 3. Results

The final cohort consisted of 700 NSTEMI patients who underwent culprit-lesion PCI. Angiographic NRF occurred in 114 patients (16.3%). Patients with NRF were older, more frequently diabetic, more likely to have Killip class II or higher, and had higher heart rates at admission. They also had longer symptom-to-angiography time, lower eGFR, higher admission glucose, higher inflammatory markers, higher peak troponin, higher platelet indices, lower hemoglobin, higher NT-proBNP, and lower LVEF. Serum total BDNF was significantly higher in patients with NRF than in patients with normal reflow [555 (465–688) vs. 386 (292–496) pg/mL, *p* < 0.001] (Table 1).

Angiographic and procedural characteristics are presented in Table 2. The NRF group had more frequent baseline TIMI 0–1 flow, high thrombus burden, multivessel disease, higher SYNTAX score, longer culprit lesions, more predilatation, longer total stent length, and more frequent bailout glycoprotein IIb/IIIa inhibitor use. Direct stenting was less frequent in the NRF group. Interobserver agreement was good for angiographic NRF diagnosis (kappa = 0.86), high thrombus burden (kappa = 0.82), and MBG grading (weighted kappa = 0.81), and excellent for SYNTAX score assessment (intraclass correlation coefficient = 0.91).

In correlation analysis, BDNF showed positive correlations with hs-CRP, WBC count, platelet count, MPV, peak hs-cTnI, NT-proBNP, SYNTAX score, lesion length, and high thrombus burden. BDNF correlated inversely with eGFR, LVEF, and baseline TIMI flow grade (Supplementary Table S2). In univariable logistic regression, BDNF was strongly associated with NRF (OR 1.78 per 100 pg/mL increase, 95% CI 1.54–2.06, *p* < 0.001). Diabetes mellitus, eGFR, hs-CRP, WBC count, NLR, platelet count, MPV, LVEF, symptom-to-angiography time, baseline TIMI 0–1 flow, high thrombus burden, SYNTAX score, and lesion length were also associated with NRF (Supplementary Table S3).

BDNF remained independently associated with NRF across all four sequential multivariable models (Table 3). The adjusted effect attenuated progressively but remained statistically significant: Model 1 OR 1.67 (95% CI 1.43–1.96), Model 2 OR 1.49 (95% CI 1.24–1.79), Model 3 OR 1.41 (95% CI 1.16–1.72), and Model 4 OR 1.31 (95% CI 1.07–1.60). In the final model, independent predictors included BDNF, lower eGFR, higher hs-CRP, lower LVEF, longer symptom-to-angiography time, baseline TIMI 0–1 flow, high thrombus burden, higher SYNTAX score, and longer lesion length.

**Figure 2:**
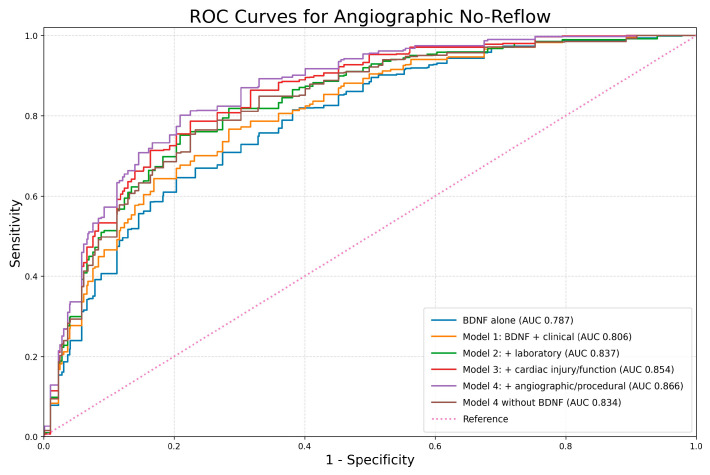
Receiver-operating characteristic curves for serum total BDNF, the four sequential multivariable models, and Model 4 without BDNF for angiographic no-reflow. AUC values shown in the legend correspond to Table 4. The dotted diagonal line represents no-discrimination performance.

ROC analysis demonstrated that BDNF alone had an AUC of 0.787 (95% CI 0.742–0.833). The optimal BDNF cut-off was 505 pg/mL, yielding 70.2% sensitivity and 74.1% specificity. This threshold was derived by the Youden index in the present dataset and should therefore be interpreted as exploratory and dataset-specific rather than as a validated clinical treatment trigger. Sequential model discrimination increased from Model 1 to Model 4. Model 4 had an AUC of 0.866 (95% CI 0.829–0.903), whereas the same final model without BDNF had an AUC of 0.834 (95% CI 0.792–0.876), resulting in a delta AUC of 0.032 (*p* = 0.005) (Table 4; Figure 2).

Calibration and internal validation supported the internal coherence of the model. Model 4 showed a Brier score of 0.085, calibration intercept of −0.02, calibration slope of 0.97, and Hosmer-Lemeshow *p* = 0.620. Bootstrap validation produced an optimism-corrected AUC of 0.852, and 10-fold cross-validation produced a mean AUC of 0.845. Addition of BDNF to the final clinical-angiographic model improved reclassification, with continuous NRI of 0.238 (95% CI 0.096–0.380; *p* = 0.001) and IDI of 0.049 (95% CI 0.025–0.073; *p* < 0.001). Decision-curve analysis suggested higher net benefit for the BDNF-enriched model across threshold probabilities of approximately 6% to 30%. In an exploratory comparison with simpler laboratory markers, the combined hs-CRP plus platelet count model yielded an AUC of 0.732, and hs-CRP plus platelet count plus MPV yielded an AUC of 0.754. Adding BDNF increased the AUC of these laboratory-marker models to 0.812 and 0.819, respectively (Supplementary Tables S4–S8 and Supplementary Figures S1 and S2).

## 4. Discussion

In this cohort of NSTEMI patients undergoing culprit-lesion PCI, admission serum total BDNF was significantly higher in patients who developed angiographic NRF and remained independently associated with NRF after sequential adjustment for clinical, laboratory, inflammatory, cardiac, angiographic, and procedural variables. The association was not limited to a crude group difference: BDNF retained significance across four progressively stricter models, and its addition to a final clinical-angiographic model improved discrimination, reclassification, overall model performance, and decision-curve net benefit.

The direction of the association requires careful interpretation. BDNF is frequently described as endothelial or vasoprotective, and this is biologically plausible. Experimental studies suggest that BDNF can support endothelial survival, modulate TrkB-dependent signaling, oppose inflammatory endothelial injury, influence nitric oxide-related pathways, and participate in angiogenic repair [8,9,10,11,23,24,25]. In chronic vascular disease, low BDNF could plausibly reflect impaired endothelial resilience, reduced repair capacity, and microvascular dysfunction. This chronic-protective framework is consistent with studies linking low BDNF or low mature BDNF to adverse vascular phenotypes [14,26].

However, ACS represents a distinct biological environment. NSTEMI and STEMI involve dynamic plaque disruption, platelet activation, thrombin generation, leukocyte recruitment, ischemia, and reperfusion injury. In this setting, serum BDNF may be influenced by platelet release and acute thrombo-inflammatory activation. Platelets store abundant BDNF and can release it during activation; therefore, serum total BDNF may behave, at least in part, as an indirect biomarker signal associated with platelet degranulation and thrombotic activity [7,12,13]. Importantly, this pathway was not directly measured in the present study because soluble platelet activation markers and platelet reactivity testing were unavailable. Thus, the platelet-release explanation should be regarded as biologically plausible and inferential rather than mechanistically proven. This is particularly relevant for PCI-related NRF, where distal embolization of platelet-rich thrombotic material, leukocyte plugging, endothelial swelling, and microvascular obstruction are central mechanisms.

The ACS data from Montone et al. are important because they help reconcile this apparent paradox. In that study, serum BDNF levels were higher in STEMI than in NSTE-ACS, and higher BDNF was associated with OCT-defined macrophage infiltration within the culprit plaque [15]. STEMI generally represents a more abrupt and occlusive thrombotic event than many NSTE-ACS presentations, with greater inflammatory and prothrombotic intensity. Therefore, higher BDNF in STEMI may reflect an acute plaque-platelet-inflammatory phenotype rather than successful vascular protection. The present NSTEMI cohort extends the same concept to a lower-grade but clinically heterogeneous ACS population in which microvascular reperfusion failure can still occur after culprit-lesion PCI.

A recent STEMI/NRF study by Zhang et al. further supports the acute-injury interpretation of BDNF [16]. That report suggested that BDNF levels were higher in STEMI patients who developed slow-flow/NRF after primary PCI and that BDNF may have a biomarker value in predicting reperfusion failure. Such a finding is pathophysiologically coherent because NRF is associated with thrombus burden, distal embolization, microvascular obstruction, inflammatory cell plugging, endothelial swelling, vasoconstriction, and larger ischemic injury. If high serum total BDNF reflects platelet activation, plaque macrophage burden, and acute tissue injury, then a positive association with NRF is plausible in STEMI and potentially also in NSTEMI.

The coronary slow-flow literature provides a complementary view. Zhang et al. reported lower serum mature BDNF in patients with the coronary slow-flow phenomenon and an inverse association between mature BDNF and mean TIMI frame count [14]. This should not be interpreted as contradicting the present findings. Coronary slow flow without obstructive epicardial disease is usually a chronic or semi-chronic microvascular/endothelial phenotype, while PCI-related NRF is an acute reperfusion failure phenotype dominated by embolization, thrombosis, inflammation, and microvascular obstruction. In addition, mature BDNF and serum total BDNF are not analytically identical, and serum total BDNF is heavily influenced by platelet release. Thus, low mature BDNF in chronic coronary slow flow and high serum total BDNF in acute thrombo-inflammatory NRF may both be biologically plausible.

The correlation pattern in the present study supports this context-dependent but inferential interpretation. BDNF correlated positively with hs-CRP, WBC count, platelet count, MPV, peak troponin, high thrombus burden, SYNTAX score, and lesion length, and negatively with eGFR, LVEF, and baseline TIMI flow. This pattern suggests that serum total BDNF is unlikely to represent a single-pathway biomarker. Rather, it may summarize a broader risk state involving inflammation, platelet-related indices, renal vulnerability, myocardial injury, impaired epicardial flow, and complex culprit anatomy. The gradual attenuation of the BDNF odds ratio from Model 1 to Model 4 indicates that part of the crude association overlaps with clinical, inflammatory, and angiographic severity, while a residual biomarker signal remains. Nevertheless, these correlations do not prove that BDNF is mechanistically specific for NRF.

The four-model design improves the interpretability of the findings. Model 1 asks whether BDNF remains associated with NRF after adjustment for basic clinical risk. Model 2 asks whether the association is independent of renal function, metabolism, inflammation, anemia, leukocyte burden, and platelet count. Model 3 tests whether BDNF remains significant after accounting for myocardial injury, ventricular dysfunction, Killip class, and symptom-to-angiography time. Model 4 then evaluates BDNF against the most direct angiographic competitors, including baseline TIMI 0–1 flow, high thrombus burden, SYNTAX score, lesion length, and multivessel disease. This hierarchy is clinically intuitive and avoids overinterpreting a biomarker before the main procedural determinants of NRF are considered.

The predictive-performance findings should be interpreted as incremental rather than diagnostic. BDNF alone had a moderate-to-good AUC of 0.787, but it should not be viewed as a stand-alone test for NRF. The more clinically relevant observation is that Model 4 with BDNF performed better than the same model without BDNF. However, the delta AUC of +0.032, although statistically significant, represents a modest incremental improvement over an already well-performing clinical-angiographic model. Therefore, the independent association of BDNF should be interpreted as statistical independence after multivariable adjustment, not as proof of clinical independence from overall disease severity or as evidence of a BDNF-specific no-reflow mechanism. The NRI, IDI, and decision-curve results support incremental risk information, but they remain hypothesis-generating and require external validation.

From a clinical research perspective, a BDNF-enriched NSTEMI NRF model may help refine preprocedural risk stratification, but it should not currently be used to mandate treatment decisions. A patient with elevated serum total BDNF, high hs-CRP, low eGFR, low LVEF, long symptom-to-angiography time, baseline TIMI 0–1 flow, and high thrombus burden may represent a higher-risk phenotype in whom operators could be more alert to possible reperfusion failure. However, the 505 pg/mL BDNF threshold was derived from the present cohort and should be considered exploratory until prospectively validated. This risk-framing role is consistent with the general principle that NRF prevention depends on identifying vulnerable coronary and microvascular substrates before reperfusion failure becomes established [1].

Several methodological points are important. BDNF measurement must be standardized because serum and plasma levels are not interchangeable and because clotting time, platelet activation, centrifugation, storage temperature, and freeze–thaw cycles influence measured concentrations [7,12,13,27]. The use of serum total BDNF is appropriate for the biological hypothesis of platelet-related acute thrombo-inflammatory activation, but it also limits direct comparison with plasma BDNF or mature BDNF studies. Similarly, NRF adjudication should be blinded to biomarker values and should include both TIMI flow and myocardial blush whenever possible.

### Limitations

First, this was a single-center prospective observational cohort study; therefore, selection bias, residual confounding, and unmeasured confounders cannot be excluded, and causality cannot be inferred. Second, serum total BDNF was measured only once on admission. Thus, temporal changes in BDNF during the acute phase, after PCI, and during recovery could not be evaluated. Third, we measured serum total BDNF and did not separately quantify mature BDNF, proBDNF, or plasma BDNF. Because serum BDNF is strongly influenced by platelet release during coagulation, the present findings may not be directly comparable with studies using plasma samples or mature BDNF-specific assays. Although the sampling, clotting, centrifugation, and storage protocols were standardized, BDNF concentrations may still be affected by pre-analytical factors, including platelet activation, storage duration, and freeze–thaw exposure. Fourth, although platelet count, MPV, and PDW were recorded, direct measures of platelet activation or thrombogenicity, such as platelet reactivity testing, soluble P-selectin, soluble CD40 ligand, or thromboxane metabolites, were not available. Therefore, platelet-derived BDNF release remains an inferential explanation based on biological plausibility and indirect associations rather than a directly demonstrated mechanism. Fifth, the exact timing and intensity of pre-procedural antiplatelet exposure could not be fully standardized in all patients, particularly with respect to P2Y12 loading relative to blood sampling. Because serum BDNF is strongly linked to platelet biology, this may have influenced measured BDNF concentrations and should be considered when interpreting the findings. Sixth, several covariates were biologically and clinically related. Although variance inflation factors were low in the final model and did not indicate severe multicollinearity, residual collinearity among inflammatory, platelet-related, myocardial injury, renal function, and angiographic severity variables may still have influenced the magnitude and interpretation of individual regression coefficients. Seventh, BDNF may be influenced by neuropsychiatric status, physical activity, sleep, metabolic factors, and several medications, including antidepressants; these variables were not systematically assessed and may have contributed to residual confounding. Eighth, comparison with simpler biomarker-only strategies was limited to exploratory analyses using routinely available inflammatory and platelet-related variables, and no external biomarker panel or direct platelet activation marker was available. Ninth, NRF was defined angiographically using the final TIMI flow and MBG. Although this approach is clinically practical and interobserver agreement for NRF and MBG was good, it does not provide direct tissue-level quantification of microvascular obstruction, and cardiac magnetic resonance imaging, invasive microvascular indices, or systematic intracoronary imaging were not available. Finally, the predictive models were internally validated, but external validation in independent multicenter NSTEMI cohorts is required before the BDNF cut-off or the BDNF-enriched model can be used for clinical decision-making. The present analysis also did not evaluate long-term clinical outcomes; therefore, whether BDNF-associated NRF translates into adverse prognosis requires further investigation.

## 5. Conclusions

Higher admission serum total BDNF was independently associated with angiographic NRF in NSTEMI patients undergoing PCI. The association remained significant across four sequential adjustment models and provided modest but statistically significant incremental risk information when added to clinical, biochemical, cardiac, and angiographic predictors. Serum total BDNF may therefore reflect a broader acute severity phenotype related to thrombo-inflammatory, platelet-associated, myocardial injury, and microvascular vulnerability pathways. Prospective multicenter validation is required before the BDNF cut-off or the BDNF-enriched model can be considered for clinical use.

## Figures and Tables

**Table 1:** Baseline clinical, laboratory, and echocardiographic characteristics according to angiographic NRF status.

Variable	Total (*n* = 700)	Reflow (*n* = 586)	NRF (*n* = 114)	*p* Value
Clinical characteristics				
Age, years	63.1 ± 10.4	62.5 ± 10.2	66.2 ± 10.8	0.001
Male sex, *n* (%)	507 (72.4)	426 (72.7)	81 (71.1)	0.724
Body mass index, kg/m^2^	28.7 ± 4.2	28.6 ± 4.1	29.1 ± 4.4	0.237
Diabetes mellitus, *n* (%)	307 (43.9)	244 (41.6)	63 (55.3)	0.007
Hypertension, *n* (%)	361 (51.6)	296 (50.5)	65 (57.0)	0.204
Dyslipidemia, *n* (%)	368 (52.6)	307 (52.4)	61 (53.5)	0.826
Current smoking, *n* (%)	268 (38.3)	225 (38.4)	43 (37.7)	0.891
Prior myocardial infarction, *n* (%)	91 (13.0)	73 (12.5)	18 (15.8)	0.329
Prior PCI, *n* (%)	128 (18.3)	103 (17.6)	25 (21.9)	0.273
Pre-admission aspirin, *n* (%)	231 (33.0)	196 (33.4)	35 (30.7)	0.568
Pre-admission statin, *n* (%)	243 (34.7)	205 (35.0)	38 (33.3)	0.731
P2Y12 loading before PCI, *n* (%)	668 (95.4)	561 (95.7)	107 (93.9)	0.387
Killip class ≥ II, *n* (%)	68 (9.7)	47 (8.0)	21 (18.4)	<0.001
Systolic blood pressure, mmHg	132 ± 22	133 ± 22	128 ± 24	0.041
Heart rate, bpm	82 ± 16	81 ± 15	88 ± 18	<0.001
Symptom-to-angiography time, h	18 (8–34)	16 (7–30)	24 (12–44)	<0.001
Laboratory and echocardiographic data				
Hemoglobin, g/dL	13.6 ± 1.5	13.7 ± 1.5	13.1 ± 1.6	<0.001
WBC, ×10^3^/µL	9.7 ± 2.8	9.4 ± 2.7	10.9 ± 3.2	<0.001
Neutrophil count, ×10^3^/µL	6.9 ± 2.6	6.6 ± 2.4	8.1 ± 3.0	<0.001
Lymphocyte count, ×10^3^/µL	1.9 (1.4–2.4)	1.9 (1.5–2.5)	1.7 (1.2–2.1)	0.002
NLR	3.5 (2.5–5.2)	3.3 (2.4–4.8)	4.8 (3.2–7.1)	<0.001
Platelet count, ×10^3^/µL	248 (208–293)	243 (205–286)	271 (227–321)	0.002
MPV, fL	10.5 ± 1.1	10.4 ± 1.0	10.9 ± 1.2	<0.001
PDW, %	13.4 ± 2.1	13.2 ± 2.0	14.3 ± 2.3	<0.001
Admission glucose, mg/dL	146 (118–201)	140 (116–193)	172 (133–236)	<0.001
HbA1c, %	6.7 ± 1.4	6.6 ± 1.3	7.1 ± 1.5	0.002
Creatinine, mg/dL	0.97 (0.82–1.15)	0.94 (0.81–1.12)	1.08 (0.91–1.32)	<0.001
eGFR, mL/min/1.73 m^2^	79.6 ± 17.4	81.3 ± 16.9	70.8 ± 18.0	<0.001
hs-CRP, mg/L	5.1 (2.8–8.2)	4.4 (2.6–7.2)	8.2 (5.0–13.1)	<0.001
Peak hs-cTnI, ng/mL	2.6 (1.2–5.0)	2.2 (1.0–4.4)	4.3 (2.2–8.0)	<0.001
NT-proBNP, pg/mL	620 (210–1500)	540 (190–1320)	1120 (420–2780)	<0.001
Total cholesterol, mg/dL	186 ± 44	185 ± 43	189 ± 46	0.356
LDL-C, mg/dL	118 ± 35	117 ± 35	121 ± 37	0.274
HDL-C, mg/dL	39 ± 10	40 ± 10	37 ± 9	0.012
Triglycerides, mg/dL	164 (114–223)	160 (112–215)	182 (128–250)	0.026
Albumin, g/dL	4.1 ± 0.4	4.2 ± 0.4	4.0 ± 0.4	<0.001
Uric acid, mg/dL	5.7 ± 1.4	5.6 ± 1.3	6.2 ± 1.5	<0.001
Serum total BDNF, pg/mL	414 (315–540)	386 (292–496)	555 (465–688)	<0.001
LVEF, %	49.6 ± 7.4	50.5 ± 6.9	45.0 ± 7.6	<0.001
Left atrial diameter, mm	37.2 ± 4.8	37.1 ± 4.8	37.9 ± 5.0	0.109

Values are mean ± SD, median (IQR), or *n* (%). BDNF: brain-derived neurotrophic factor; eGFR: estimated glomerular filtration rate; HDL-C: high-density lipoprotein cholesterol; hs-CRP: high-sensitivity C-reactive protein; hs-cTnI: high-sensitivity cardiac troponin I; LDL-C: low-density lipoprotein cholesterol; LVEF: left ventricular ejection fraction; MPV: mean platelet volume; NLR: neutrophil-to-lymphocyte ratio; NRF: no-reflow; NT-proBNP: N-terminal pro-B-type natriuretic peptide; PCI: percutaneous coronary intervention; PDW: platelet distribution width; WBC: white blood cell count.

**Table 2:** Angiographic and procedural characteristics according to angiographic NRF status.

Variable	Total (*n* = 700)	Reflow (*n* = 586)	NRF (*n* = 114)	*p* Value
Radial access, *n* (%)	620 (88.6)	525 (89.6)	95 (83.3)	0.053
Culprit LAD, *n* (%)	325 (46.4)	267 (45.6)	58 (50.9)	0.300
Culprit RCA, *n* (%)	219 (31.3)	187 (31.9)	32 (28.1)	0.420
Baseline TIMI 0–1, *n* (%)	171 (24.4)	120 (20.5)	51 (44.7)	<0.001
Baseline TIMI 2, *n* (%)	211 (30.1)	180 (30.7)	31 (27.2)	0.451
High thrombus burden, *n* (%)	113 (16.1)	74 (12.6)	39 (34.2)	<0.001
Multivessel disease, *n* (%)	353 (50.4)	287 (49.0)	66 (57.9)	0.080
SYNTAX score	16 (11–21)	15 (11–20)	20 (15–25)	<0.001
Lesion length, mm	24.5 ± 7.2	23.7 ± 6.8	28.6 ± 8.5	<0.001
Reference vessel diameter, mm	3.0 ± 0.4	3.0 ± 0.4	2.9 ± 0.4	0.087
Direct stenting, *n* (%)	279 (39.9)	249 (42.5)	30 (26.3)	0.001
Predilatation, *n* (%)	296 (42.3)	236 (40.3)	60 (52.6)	0.014
Total stent length, mm	29 (22–38)	28 (22–36)	35 (26–45)	<0.001
Post-dilatation, *n* (%)	294 (42.0)	244 (41.6)	50 (43.9)	0.657
Rescue GP IIb/IIIa inhibitor, *n* (%)	73 (10.4)	52 (8.9)	21 (18.4)	0.002
Intracoronary vasodilator use, *n* (%)	86 (12.3)	54 (9.2)	32 (28.1)	<0.001

Values are mean ± SD, median (IQR), or *n* (%). GP: glycoprotein; LAD: left anterior descending artery; NRF: no-reflow; RCA: right coronary artery; TIMI: thrombolysis in myocardial infarction.

**Table 3:** Sequential multivariable logistic regression models for angiographic NRF.

Predictor	Model 1 OR (95% CI), *p*	Model 2 OR (95% CI), *p*	Model 3 OR (95% CI), *p*	Model 4 OR (95% CI), *p*
BDNF, per 100 pg/mL	1.67 (1.43–1.96), <0.001	1.49 (1.24–1.79), <0.001	1.41 (1.16–1.72), 0.001	1.31 (1.07–1.60), 0.009
Age, per 10 years	1.21 (0.98–1.49), 0.073	1.16 (0.93–1.45), 0.184	1.12 (0.89–1.40), 0.333	1.09 (0.86–1.38), 0.469
Male sex	0.94 (0.58–1.52), 0.806	0.96 (0.57–1.61), 0.877	0.98 (0.58–1.66), 0.939	0.99 (0.58–1.70), 0.976
Diabetes mellitus	1.55 (1.00–2.41), 0.049	1.36 (0.84–2.20), 0.209	1.30 (0.79–2.15), 0.302	1.25 (0.75–2.09), 0.392
Hypertension	1.12 (0.71–1.77), 0.628	1.07 (0.66–1.73), 0.778	1.05 (0.64–1.72), 0.843	1.03 (0.62–1.70), 0.920
Smoking status	0.97 (0.61–1.55), 0.906	0.95 (0.58–1.56), 0.842	0.94 (0.57–1.56), 0.816	0.95 (0.56–1.61), 0.846
BMI, per 1 kg/m^2^	1.02 (0.97–1.08), 0.405	1.01 (0.96–1.07), 0.660	1.01 (0.95–1.07), 0.793	1.00 (0.94–1.07), 0.922
eGFR, per 10 mL/min lower	-	1.28 (1.07–1.52), 0.006	1.23 (1.03–1.48), 0.024	1.18 (1.00–1.41), 0.050
Admission glucose, per 20 mg/dL	-	1.07 (0.99–1.16), 0.096	1.05 (0.96–1.15), 0.265	1.03 (0.94–1.14), 0.503
hs-CRP, per ln-unit	-	1.50 (1.16–1.94), 0.002	1.41 (1.08–1.84), 0.012	1.30 (1.01–1.69), 0.041
WBC, per 1 × 10^3^/µL	-	1.05 (0.98–1.12), 0.142	1.04 (0.96–1.11), 0.324	1.03 (0.96–1.11), 0.381
Hemoglobin, per 1 g/dL lower	-	1.11 (0.94–1.31), 0.217	1.08 (0.91–1.29), 0.378	1.06 (0.88–1.28), 0.531
Platelet count, per 50 × 10^3^/µL	-	1.12 (0.96–1.31), 0.149	1.08 (0.92–1.27), 0.343	1.05 (0.89–1.24), 0.563
LVEF, per 5% lower	-	-	1.32 (1.10–1.58), 0.003	1.22 (1.01–1.48), 0.037
Peak hs-cTnI, per ln-unit	-	-	1.18 (0.97–1.44), 0.096	1.11 (0.90–1.38), 0.330
Killip class ≥ II	-	-	1.41 (0.76–2.62), 0.276	1.29 (0.68–2.45), 0.438
Symptom-to-angiography time, per 6 h	-	-	1.11 (1.03–1.20), 0.006	1.08 (1.00–1.17), 0.038
Baseline TIMI 0–1	-	-	-	1.82 (1.07–3.10), 0.027
High thrombus burden	-	-	-	2.21 (1.25–3.92), 0.007
SYNTAX score, per 5 points	-	-	-	1.18 (1.00–1.40), 0.046
Lesion length, per 5 mm	-	-	-	1.19 (1.03–1.38), 0.021
Multivessel disease	-	-	-	1.12 (0.67–1.87), 0.663

Model 1: BDNF plus clinical variables. Model 2: Model 1 plus laboratory and inflammatory variables. Model 3: Model 2 plus cardiac injury, cardiac functional variables, and clinical timing represented by symptom-to-angiography time. Model 4: Model 3 plus angiographic and procedural variables. BDNF: brain-derived neurotrophic factor; BMI: body mass index; CI: confidence interval; eGFR: estimated glomerular filtration rate; hs-CRP: high-sensitivity C-reactive protein; hs-cTnI: high-sensitivity cardiac troponin I; LVEF: left ventricular ejection fraction; OR: odds ratio; TIMI: thrombolysis in myocardial infarction.

**Table 4:** ROC-derived and incremental predictive performance of BDNF-enriched models.

Model/Index	AUC (95% CI)	Optimal Threshold	Sensitivity (%)	Specificity (%)	Brier Score	Delta AUC	*p* Value
BDNF alone	0.787 (0.742–0.833)	505 pg/mL	70.2	74.1	0.110	-	-
Model 1	0.806 (0.762–0.850)	Predicted risk ≥ 0.15	72.8	76.5	0.104	Reference	-
Model 2	0.837 (0.797–0.877)	Predicted risk ≥ 0.16	75.4	79.1	0.095	+0.031	0.018
Model 3	0.854 (0.815–0.893)	Predicted risk ≥ 0.16	77.2	80.5	0.089	+0.017	0.049
Model 4 without BDNF	0.834 (0.792–0.876)	Predicted risk ≥ 0.17	71.1	79.0	0.093	Reference	-
Model 4 with BDNF	0.866 (0.829–0.903)	Predicted risk ≥ 0.16	79.0	82.6	0.085	+0.032 vs. Model 4 without BDNF	0.005

AUC: area under the curve; BDNF: brain-derived neurotrophic factor; ROC: receiver-operating characteristic. Delta AUC values for Models 2 and 3 represent the incremental change versus the immediately preceding sequential model. Model 4 with BDNF versus Model 4 without BDNF was used to evaluate the incremental predictive value of serum total BDNF beyond clinical, biochemical, cardiac, angiographic, and procedural predictors. The BDNF-only cut-off was derived by the Youden index and should be interpreted as exploratory.

## Data Availability

The data underlying the current study are available from the corresponding author upon reasonable request.

## Supplementary Materials

The following supporting information can be downloaded at: https://www.mdpi.com/article/10.3390/medicina62071211/s1, Table S1. NRF phenotype and BDNF quartile gradient. Table S2. Spearman correlation between serum total BDNF and selected parameters. Table S3. Univariable logistic regression for angiographic NRF. Table S4. Calibration and internal validation of the final BDNF-enriched Model 4. Table S5. Incremental value of adding BDNF to clinical and clinical-angiographic models. Table S6. Sensitivity and robustness analyses for BDNF as a predictor of NRF. Table S7. Multicollinearity diagnostics and missing-data summary for the final model. Table S8. Exploratory comparison of BDNF with simple laboratory-marker models. Figure S1. Calibration plot for Model 4. Points represent observed NRF rates across deciles of predicted probability. Figure S2. Decision-curve analysis comparing Model 4 with BDNF, Model 4 without BDNF, BDNF alone, treat-all, and treat-none strategies.

## Abbreviations

ACS: acute coronary syndrome; AUC: area under the curve; BDNF: brain-derived neurotrophic factor; BMI: body mass index; CABG: coronary artery bypass grafting; CI: confidence interval; eGFR: estimated glomerular filtration rate; ELISA: enzyme-linked immunosorbent assay; GP: glycoprotein; hs-CRP: high-sensitivity C-reactive protein; hs-cTnI: high-sensitivity cardiac troponin I; IDI: integrated discrimination improvement; LAD: left anterior descending artery; LVEF: left ventricular ejection fraction; MBG: myocardial blush grade; MPV: mean platelet volume; NLR: neutrophil-to-lymphocyte ratio; NRI: net reclassification improvement; NRF: no-reflow; NSTEMI: non-ST-segment elevation myocardial infarction; NT-proBNP: *N*-terminal pro-B-type natriuretic peptide; OCT: optical coherence tomography; OR: odds ratio; PCI: percutaneous coronary intervention; PDW: platelet distribution width; RCA: right coronary artery; ROC: receiver-operating characteristic; STEMI: ST-segment elevation myocardial infarction; TIMI: thrombolysis in myocardial infarction; VIF: variance inflation factor; WBC: white blood cell count.
