# Supplementary material for: Admission Serum Total Brain-Derived Neurotrophic Factor and Angiographic No-Reflow in Non-ST-Segment Elevation Myocardial Infarction Undergoing Percutaneous Coronary Intervention
Source: Medicina (Kaunas). 2026 Jun 23;62(7):1211. doi: 10.3390/medicina62071211 (PMC13413712; doi:10.3390/medicina62071211)
Supplement: Supplementary file 1 [file medicina-62-01211-s001.zip › medicina-4369168-supplementary.pdf]

Supplementary Materials

Table S1. NRF phenotype and BDNF quartile gradient.

Item	Category	n/N (%) or value
Primary endpoint	Any angiographic NRF	114/700 (16.3)
Final TIMI flow in NRF cases	TIMI 0-1	14/114 (12.3)
Final TIMI flow in NRF cases	TIMI 2	59/114 (51.8)
Final TIMI flow in NRF cases	TIMI 3 with MBG 0-1	41/114 (36.0)
BDNF quartile 1	<=310 pg/mL	12/175 (6.9)
BDNF quartile 2	311-407 pg/mL	19/175 (10.9)
BDNF quartile 3	408-532 pg/mL	30/175 (17.1)
BDNF quartile 4	>532 pg/mL	53/175 (30.3)
Trend test	Across BDNF quartiles	p < 0.001

Table S2. Spearman correlation between serum total BDNF and selected parameters.

Variable	Spearman r	p value
Age	0.08	0.041
eGFR	-0.20	<0.001
WBC	0.20	<0.001
NLR	0.23	<0.001
hs-CRP	0.32	<0.001
Peak hs-cTnI	0.27	<0.001
NT-proBNP	0.25	<0.001
Platelet count	0.17	<0.001
MPV	0.21	<0.001
PDW	0.18	<0.001
LVEF	-0.35	<0.001
Baseline TIMI flow grade	-0.30	<0.001
SYNTAX score	0.19	<0.001
Lesion length	0.16	<0.001
High thrombus burden	0.24	<0.001

Table S3. Univariable logistic regression for angiographic NRF.

Variable	Unit/category	OR	95% CI	p value
BDNF	per 100 pg/mL increase	1.78	1.54-2.06	<0.001
Age	per 10-year increase	1.35	1.11-1.63	0.002
Male sex	yes vs. no	0.92	0.59-1.43	0.724
Diabetes mellitus	yes vs. no	1.74	1.16-2.60	0.007
Hypertension	yes vs. no	1.30	0.87-1.94	0.205
BMI	per 1 kg/m2	1.03	0.98-1.08	0.238
Symptom-to-angiography time	per 6 h increase	1.16	1.08-1.25	<0.001
eGFR	per 10 mL/min decrease	1.43	1.24-1.65	<0.001
hs-CRP	per ln-unit increase	1.74	1.41-2.14	<0.001
WBC	per 1 x10^3/uL increase	1.17	1.09-1.26	<0.001
NLR	per ln-unit increase	1.66	1.32-2.08	<0.001
Hemoglobin	per 1 g/dL decrease	1.26	1.10-1.45	0.001
Platelet count	per 50 x10^3/uL increase	1.23	1.08-1.40	0.002
MPV	per 1 fL increase	1.45	1.18-1.78	<0.001
LVEF	per 5% decrease	1.61	1.39-1.87	<0.001
Peak hs-cTnI	per ln-unit increase	1.35	1.16-1.58	<0.001
Baseline TIMI 0-1	yes vs. no	3.14	2.04-4.83	<0.001
High thrombus burden	yes vs. no	3.62	2.28-5.75	<0.001
SYNTAX score	per 5-point increase	1.47	1.27-1.71	<0.001
Lesion length	per 5-mm increase	1.45	1.28-1.65	<0.001
Multivessel disease	yes vs. no	1.43	0.96-2.13	0.080

Table S4. Calibration and internal validation of the final BDNF-enriched Model 4.

Validation method	Statistic	Value
Apparent performance	AUC	0.866
Apparent performance	Brier score	0.085
Apparent performance	Calibration intercept	-0.02
Apparent performance	Calibration slope	0.97
Hosmer-Lemeshow test	chi-square (df=8), p	6.28, p=0.620
Bootstrap, 1000 resamples	Mean optimism	0.014
Bootstrap, 1000 resamples	Optimism-corrected AUC	0.852
Bootstrap, 1000 resamples	Optimism-corrected calibration slope	0.92
Bootstrap, 1000 resamples	Optimism-corrected Brier score	0.090
10-fold cross-validation	Mean AUC	0.845
10-fold cross-validation	AUC range across folds	0.809-0.876
10-fold cross-validation	Mean calibration slope	0.90
10-fold cross-validation	Mean Brier score	0.092
Shrinkage estimate	Uniform shrinkage factor	0.92

Table S5. Incremental value of adding BDNF to clinical and clinical-angiographic models.

Comparison	Delta AUC	p value	Continuous NRI (95% CI)	p value	IDI (95% CI)	p value
Model 4 with BDNF vs Model 4 without BDNF	+0.032	0.005	0.238 (0.096-0.380)	0.001	0.049 (0.025-0.073)	<0.001
BDNF + clinical model vs clinical model alone	+0.087	<0.001	0.326 (0.178-0.474)	<0.001	0.066 (0.038-0.094)	<0.001

Table S6. Sensitivity and robustness analyses for BDNF as a predictor of NRF.

Analysis	BDNF effect estimate	95% CI	p value	Comment
BDNF per 100 pg/mL in Model 4	OR 1.31	1.07-1.60	0.009	Primary analysis
BDNF log-transformed	OR 1.82	1.20-2.75	0.004	Consistent after skewness adjustment
BDNF highest quartile vs lower three quartiles	OR 2.01	1.14-3.54	0.016	Threshold-like gradient
Replacing hs-CRP with NLR	OR 1.32	1.08-1.61	0.007	Stable alternative inflammatory adjustment
Adding MPV to the final model	OR 1.29	1.05-1.59	0.016	Not fully explained by platelet indices
Excluding patients with rescue GP IIb/IIIa use	OR 1.28	1.03-1.59	0.026	Not driven by bailout pharmacology
Excluding baseline TIMI 0-1 culprit lesions	OR 1.27	1.00-1.61	0.049	Persists in non-occlusive baseline flow
Using final TIMI <3 only as endpoint	OR 1.36	1.06-1.74	0.015	Robust with stricter endpoint
Replacing SYNTAX score with multivessel disease	OR 1.33	1.09-1.63	0.005	Stable alternative anatomic adjustment
Complete-case analysis only	OR 1.30	1.06-1.60	0.012	Similar to primary analysis

Table S7. Multicollinearity diagnostics and missing-data summary for the final model.

Variable	Tolerance	VIF	Missing, n (%)
BDNF	0.80	1.25	0 (0.0)
Age	0.84	1.19	0 (0.0)
Diabetes mellitus	0.87	1.15	0 (0.0)
eGFR	0.76	1.32	2 (0.3)
hs-CRP	0.70	1.43	18 (2.6)
WBC	0.72	1.39	0 (0.0)
Hemoglobin	0.88	1.14	0 (0.0)
Platelet count	0.84	1.19	0 (0.0)
LVEF	0.74	1.35	9 (1.3)
Peak hs-cTnI	0.78	1.28	5 (0.7)
Symptom-to-angiography time	0.82	1.22	4 (0.6)
Baseline TIMI 0-1	0.79	1.27	0 (0.0)
High thrombus burden	0.77	1.30	0 (0.0)
SYNTAX score	0.68	1.47	0 (0.0)
Lesion length	0.70	1.43	0 (0.0)

Table S8. Exploratory comparison of BDNF with simple laboratory-marker models.

Model	AUC (95% CI)	Delta AUC	p value	Sensitivity (%)	Specificity (%)
hs-CRP + platelet count	0.732 (0.681-0.783)	Reference	-	66.7	69.8
hs-CRP + platelet count + BDNF	0.812 (0.770-0.854)	+0.080	<0.001	73.7	77.0
hs-CRP + platelet count + MPV	0.754 (0.704-0.803)	Reference	-	68.4	70.6
hs-CRP + platelet count + MPV + BDNF	0.819 (0.778-0.860)	+0.065	<0.001	74.6	77.8
BDNF alone	0.787 (0.742-0.833)	-	-	70.2	74.1

AUC: area under the curve; BDNF: brain-derived neurotrophic factor; hs-CRP: high-sensitivity C-reactive protein; MPV: mean platelet volume. These analyses were exploratory and were performed to evaluate whether the BDNF signal duplicated simple inflammatory and platelet-related laboratory information.

Supplementary Figures

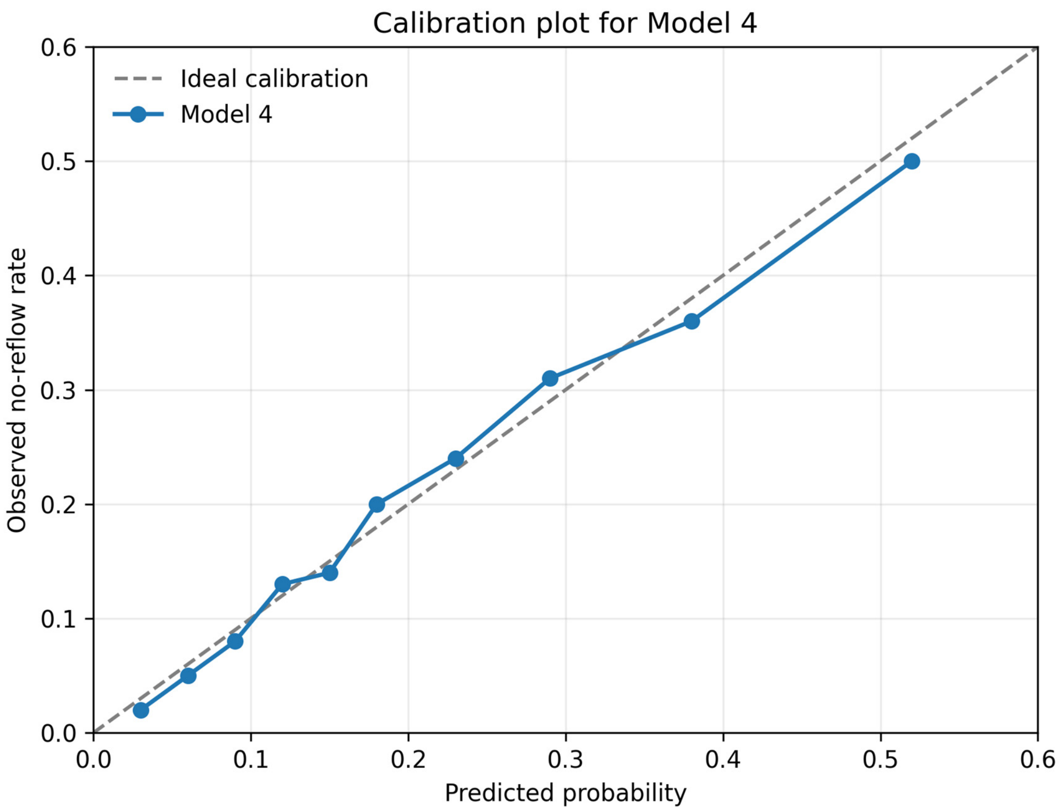

Figure S1. Calibration plot for Model 4. Points represent observed NRF rates across deciles of predicted probability.

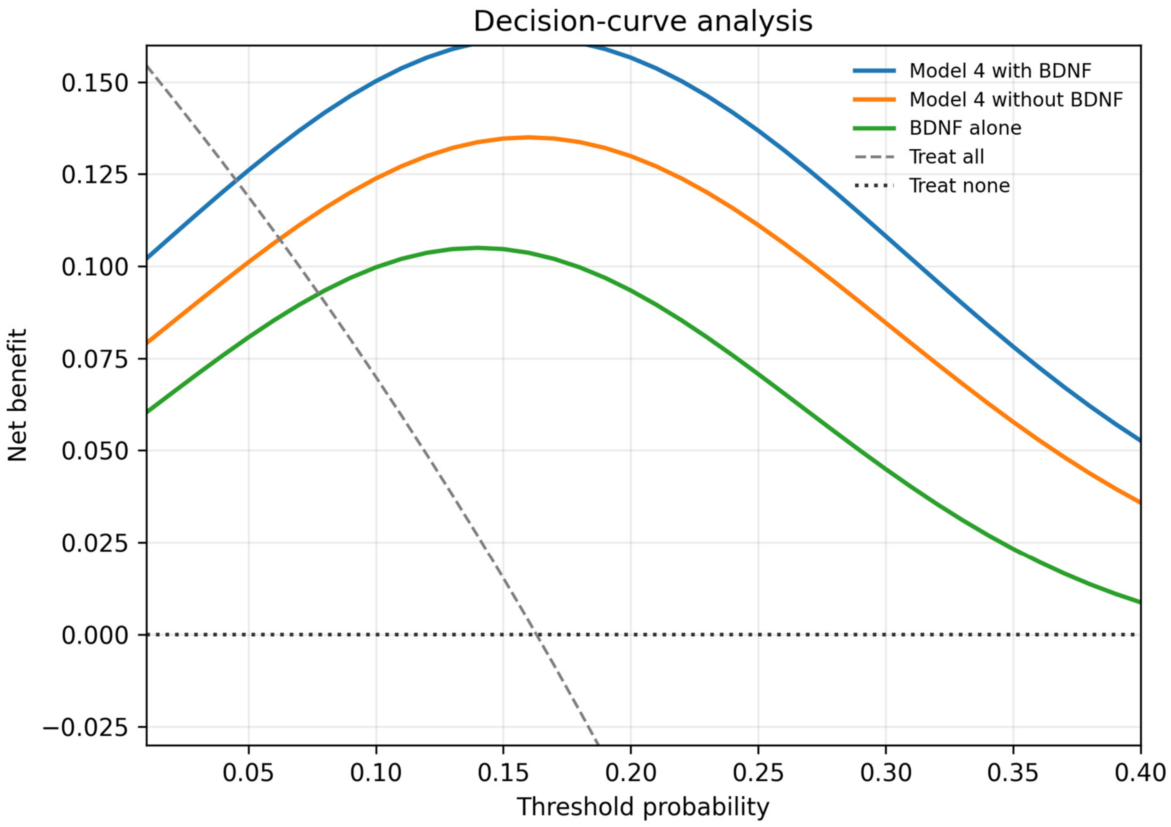

Figure S2. Decision-curve analysis comparing Model 4 with BDNF, Model 4 without BDNF, BDNF alone, treat-all, and treat-none strategies.
